# A Conversation with Outgoing ASTMH President Julie Jacobson

**DOI:** 10.4269/ajtmh.21-interview

**Published:** 2022-03-09

*Julie Jacobson, MD, DTM&H, recently completed her term as the American Society of Tropical Medicine and Hygiene (ASTMH) President. Dr. Jacobson is a global health leader and physician with a life-long commitment to addressing complex public health problems. She has worked at the CDC and the Bill & Melinda Gates Foundation, and currently serves as Managing Partner for the global health non-profit Bridges to Development. She recently sat down with science writer Matthew Davis to reflect on the past year, including a discussion of how her commitment to “courage, compassion, and culture”—the theme of her presidency—was especially relevant as the COVID-19 pandemic continued to generate novel challenges.*

## What was it like serving as ASTMH President almost 2 years into a global health crisis?

It’s been an incredible year—and I know we all feel this way.

When this pandemic first began, I think many of us felt like we knew what we were up against, that there was a sense of, “Ok, we’ve got this and we’ll rally.” But the lived experience has been very different. At a personal and professional level, the pandemic just keeps creating little fires everywhere and our resilience has been truly tested. For example, we all talked about how we can work together virtually, and we really have. But you can also enter this sort of time-zone-free existence where you struggle to create boundaries. There are just no rules of engagement with this kind of situation.

I think many people are now struggling with being burned out and wondering how to show up every day with their best self. It’s been a learning process to find new ways of coping and developing resiliency.

## Your stated themes at the start of your presidency were “courage, compassion, and culture.” How do you view them now that a year has passed?

I think they became more and more relevant as the year progressed. Courage is, of course, something that has been enormously important during the pandemic. When you saw people in our world thrust into the spotlight, like (former CDC director) Bill Foege or Tony Fauci, they may not have asked for it, but they had the courage to accept the role and speak the truth.

I’m sure such moments happen every day for members of our Society. They are being asked “Are these vaccines really needed?” or “Is COVID-19 really that serious?” Even if it’s just people in your family or community or at the grocery store, you have to have the courage to engage.

Compassion was one I was thinking about initially when discussing the presidency because it is such a huge driver behind the work we do. It’s important to recognize and honor that. Our collective experience with the pandemic also has reminded all of us of the importance of compassion in our everyday personal and professional interactions as well. We can see the toll this ordeal is taking on our friends, colleagues, and family members; on our kids in school who may be struggling. So it has become that much more important to have compassion for everyone in our lives.

The importance of culture also has come through during the events of the past year, whether it’s in the context of COVID-19 or issues related to climate change or racism or nationalism. One’s cultural background and perspectives affect how we experience and process these events. It’s also a reminder of the strength we as a scientific society gain from our cultural diversity. We have members from 115 different countries all bringing something different and valuable to the table.

## How did your year as President change—or did it change—as a result of the global pandemic?

**Figure:**
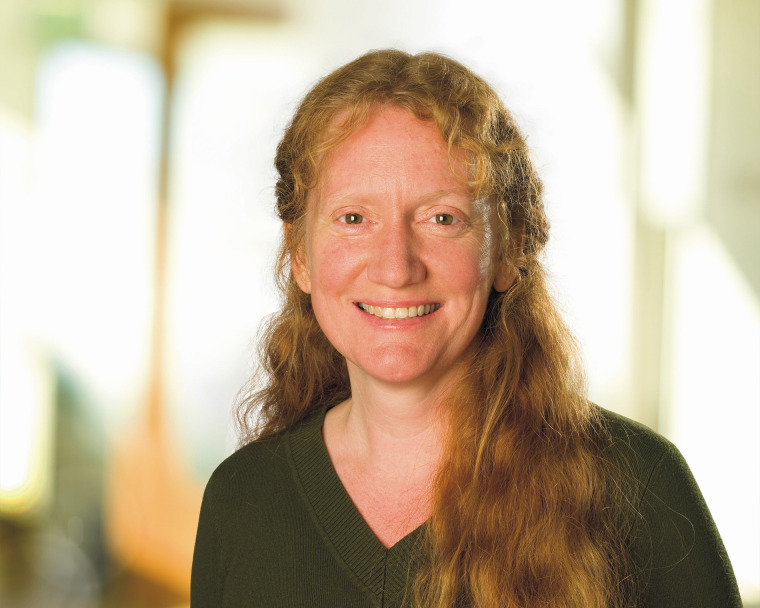
2021 ASTMH President Julie Jacobson, MD, DTM&H, FASTMH

I had to look for more opportunities to engage our community. One of the things I did was to develop a series of fireside chats to help provide something inspiring. I spoke with Soumya Swaminathan about her experience as WHO’s Chief Scientist, and with Bill Foege about the importance of courage, compassion, and culture. And I recently had a chat over e-mail with former President Jimmy Carter. They were all so inspiring. And I wanted to provide something inspiring because much of what we are hearing today is not inspiring.

I also learned that we can discover new ways of connecting to people in our community, that we can engage with people in different settings around the world more than we thought was possible. Hopefully, that will make our Society more robust going forward, because we will continue these new ways of interreacting.

But I also learned that there is nothing that replaces getting together in person. I will confess that when we had to cancel the in-person meeting this year, it was pretty devastating for me. It made me realize how much I was longing for that face-to-face interaction, even those hallway conversations, because they actually can add a lot to our work.

## This year, the United Kingdom announced severe cuts in funding for Neglected Tropical Diseases (NTDs). How did you engage ASTMH in response to this?

It was a devastating turn for the communities that suffer from these diseases and for the work involving out Society’s members. It was like losing 50% of global funding for NTDs. We did get involved and used our voice to highlight the importance of these investments and to think about creative problem solving in the wake of the decision.

The decision is not going to change, but it was an important moment for reaching out to our colleagues in the Royal Society of Tropical Medicine and Hygiene in the United Kingdom. We want them and all our valued colleagues in the United Kingdom or supported by funds from the United Kingdom to know we support them and we will continue to value their partnership.

## Looking to the future, how do you see the pandemic continuing to shape the world of global health and the control of infectious disease?

I have concerns, but also hopes. One of my biggest concerns for the future is over the significant amount of anti-science and anti-vaccine messages that have become such a constant during the pandemic. How do we fix that? How do we build back trust?

I’m especially concerned because vaccines are supposed to be how the world gets out of this pandemic. But when they become more widely available, how will their impact be affected by the globalization of anti-vaccine misinformation? I was recently on a call with colleagues in Papua New Guinea. Vaccines are becoming available there but are facing high levels of vaccine refusal because of misinformation that has made it around the world through different channels. I worry this is going to be a repeated story in different parts of the world. It’s a huge tragedy and causing harm to vulnerable populations that we try to support.

Going forward, we will need to learn more about how information flows, and to create strategies for effectively countering this mistrust by communicating more effectively with different populations around the world, because misinformation is getting out very quickly.

In terms of hopes, I am optimistic that as we open up more and are able to spend more time together, we will take the lessons we have learned from the pandemic and start to apply them. For example, we have talked in the world of health care about the importance of integrating approaches and not thinking about disease in such vertical ways. If we are going to rebound from this situation and rebuild our health system, we need to be thinking more holistically about health, not just about interventions for single diseases. Maybe doing so can help break down silos and funding disparities among different programs.

For a start, we have found that we must put communities at the center of health care, and we need to think more about how to do that and learn from their innovation. We have to listen to and understand the needs and concerns of people in different communities. They will tell us how to bring back trust.

Editor’s Note: This interview was first published on the 2021 ASTMH Annual Meeting blog.

